# Self-Adhesive and Reprocessable Ionogel Sensor from Controllable Ionized Corncob Cellulose

**DOI:** 10.3390/polym17070921

**Published:** 2025-03-28

**Authors:** Jialin Jian, Jiaqi Su, Yujian Song, Jingshun Wang, Jie Cong, Shuangying Wei, Zhenhua Gao, Shuaiyuan Han

**Affiliations:** 1 College of Material Science and Engineering, Northeast Forestry University, Harbin 150040, China; 2 Engineering Research Center of Advanced Wooden Materials, Ministry of Education, Northeast Forestry University, Harbin 150040, China; 3 State Key Laboratory of Utilization of Woody Oil Resource, Northeast Forestry University, Harbin 150040, China; 4 Key Laboratory of Biobased Material Science and Technology, Ministry of Education, Northeast Forestry University, Harbin 150040, China

**Keywords:** corncob cellulose, ATRP, ionogel, sensor

## Abstract

In recent years, the disposal of agricultural lignocellulosic residues has been accompanied by problems such as resource waste and environmental pollution. Therefore, the development of valorization technologies has emerged as a strategic priority in sustainable materials science. This study pioneered the use of corncob cellulose as the substrate (a representative agricultural lignocellulosic residue) and transformed it into ionized cellulose by grafting methacryloxyethyl trimethyl ammonium chloride (DMC) via atom transfer radical polymerization (ATRP) and UV-initiated polymerization. Characterizations demonstrated exceptional properties: robust mechanical strength (1.28 MPa tensile strength with 573% elongation); outstanding thermal stability (stable to 278 °C); cryogenic tolerance (retaining flexibility at −25 °C); and universal adhesion capability (4.23 MPa to glass substrates, with adequate interfacial bonding across diverse surfaces). Meanwhile, the ionogel exhibited exceptional sensing sensitivity (gauge factor, GF = 1.23–2.08), demonstrating versatile application potential in wearable electronics. It achieved the precise detection of subtle strains (1–5% strain range) and the high-fidelity acquisition of electrocardiogram (ECG) signals. This study broadens the design paradigm of agricultural lignocellulosic residue-based functional materials. It also provides a novel technical pathway to develop eco-friendly intelligent sensors.

## 1. Introduction

The agricultural and forestry sectors generate significant quantities of annual biomass waste through production and industrial operations. Conventional disposal approaches result in resource underutilization. Landfilling and incineration are accompanied by the generation of pollutants. Byproducts (e.g., methane or other greenhouse gases) and combustion residues (notably dioxin contaminants) can pose a threat to the environment. Corncob is a representative agricultural residue, which is mainly used as a low-grade fuel. It contains 32–36% cellulose, 35–40% hemicellulose, and ~25% lignin. Cellulose is a stereoregular polymer consisting of β-(1,4)-glycosidic-bonded D-glucose units, which has emerged as an attractive biodegradable biopolymer [1,2,3,4]. Despite the fact that cellulose has been utilized in papermaking, food additives, pharmaceuticals, tissue engineering [5], and sensing technologies [6,7,8,9], poor compatibility with synthetic polymers caused by its high crystallinity and solubility defects have seriously restricted its functional development.

To overcome the above limitations, research currently focuses on chemical modification strategies (esterification, sulfonation, etherification, and graft copolymerization). ATRP has gained prominence in cellulose modification due to its precise control over polymerization kinetics. Cellulose-initiated ATRP enables precision grafting modifications to optimize solubility and interfacial compatibility. For example, Ou et al. developed antibacterial wound dressings by grafting dimethylaminoethyl methacrylate (DMAEMA) onto bacterial cellulose via ATRP followed by quaternization [10]. Vargas et al. synthesized corncob cellulose-grafted polymers with 4-vinylpyridine for heavy metal adsorption [11].

In recent years, stretchable flexible sensors have received a great deal of attention from researchers, due to their stable electrical properties under complex deformation [12,13]. Adhesive gels are studied as a new type of flexible electronics, due to their skin conformability, ultra-stretchability, and self-healing properties [14]. They have potential in biomedicine, motion detection [15], human–machine interfaces [16], and beyond. Biomass-derived sensors are particularly attractive due to their biocompatibility, environmental sustainability, low toxicity, and cost-effectiveness. Some innovations include Tie et al.’s highly conductive and robust composite hydrogel combining polypyrrole/cellulose nanofibrils with polyvinyl alcohol and phytic acid [17], and Liu et al.’s interlocked cellulose ionogel sensor enabling dual-mode temperature/pressure sensing [18].

Conventional hydrogel sensors frequently exhibit dehydration and poor long-term stability. Some hydrogels require additional water-retaining encapsulation, or additional conductive media for sensing [19]. Therefore, this paper used polymer electrolyte and ionized cellulose to prepare ionogels, which were mainly constructed by electrostatic interaction. Compared with the small-molecule electrolyte, the macromolecule ion electrolyte in the gels had excellent stability. It was not readily lost from the system. Physical interactions in the ionogels gave the gel recyclable and self-healing properties, which prolonged its service life. However, compared with chemical gels, the mechanical properties of physical gels (such as tensile strength) need to be improved [20].

After the grafting modification of cellulose, the ionized cellulose with lamellar structures can effectively improve the mechanical properties of the gel. This study provided a new solution to improve the long-term use of sensing materials. At the same time, this paper directly extracted and modified bio-based materials from agricultural and forestry wastes. The purpose was to replace part of the petroleum-based feedstock, which exhibited an effective way to prepare environmental-friendly sensing materials. And cellulose-based strain sensors play a crucial role in enhancing the sensitivity of conductive polymer composites. Fu et al. used bamboo fiber as a backbone to prepare pressure sensors with high sensing sensitivity and ultra-wide working range [21,22].

**Figure 1:**
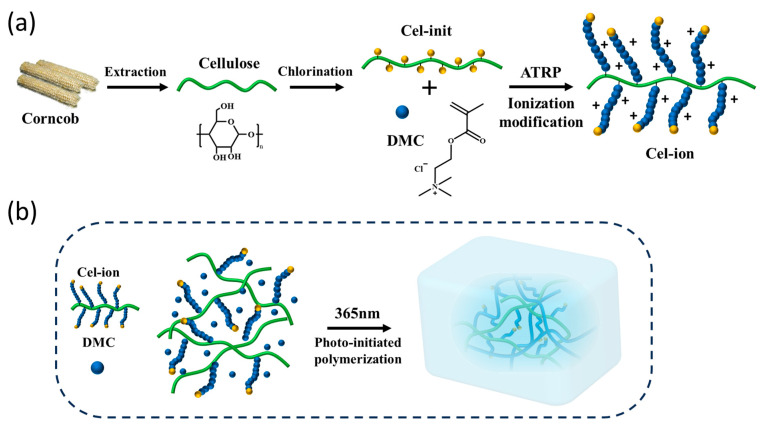
Schematic diagram for the preparation of (**a**) ionized cellulose and (**b**) cellulose cation/PDMC ionogel.

Building upon these advancements in cellulose modification and ionogel sensor, this study innovatively integrates ATRP-grafted cellulose with ionic liquid monomers (DMCs), to fabricate ionogels for sensing applications via UV-initiated polymerization (Figure 1). The system exhibits three distinct advantages: (1) electrostatic interactions confer robust adhesion (4.23 MPa bonding strength to glass) and intrinsic self-healing capability; (2) ionic interactions and hydrogen bonding interactions between PDMC and ionized cellulose act as physical crosslinking in ionic gels, this linear topological structures enable recyclability and reprocessability; and (3) polyionic conductive networks endow the gel with exceptional strain sensitivity (GF = 1.23–2.08) and rapid response (0.221–0.322 s). Successful application examples in strain testing (1–60% strain) and high-fidelity ECG signal acquisition demonstrate its practical viability, establishing a novel paradigm for functional biomaterial design.

## 2. Materials and Methods

### 2.1. Materials

Corncob (Suyu 28) was purchased from Surui Straw Processing Plant Co. (Lianyungang, China). Sodium hydroxide (NaOH, 99.7%), glacial acetic acid (99.7%), and *N*,*N*-dimethylformamide (DMF, 99.7%) were purchased from Tianjin Tianli Chemical Reagent Co., Ltd. (Tianjin, China). Sodium chlorite (NaClO_2_, 99.7%) was purchased from Tianjin Damao Chemical Reagents Partnership (Tianjin, China). Anhydrous ethanol (99.7%) was purchased from Tianjin Fuyu Fine Chemical Co., Ltd. (Tianjin, China). Acetone (99.7%) was purchased from Shanghai Hushi Laboratory Equipment Co., Ltd. (Shanghai, China). Anhydrous lithium chloride (LiCl, 99%+), *N*,*N*-dimethylacetamide (DMAc, ultradry, 99.8%), 2-chloropropionyl chloride (98%), *N*,*N*,*N*′,*N*″,*N*″-pentamethyldiethylenetriamine (PMDETA, 98%), cuprous bromide (CuBr, 99%), methacryloyloxyethyltrimethylammonium chloride (DMC, 79–81%), and 2-hydroxy-4′-(2-hydroxyethoxy)-2-methylpropiophenone (photoinitiator 2959, 98%) were purchased from Shanghai Adamas Reagent Co., Ltd. (Shanghai, China).

### 2.2. Extraction of Corncob Cellulose

Corncob fragments were mechanically activated using a planetary ball mill (200 rpm; bidirectional rotation; 1 h per direction) to maximize surface area exposure. In total, 10 g of milled powder was added to 500 mL of 7 wt% NaOH aqueous solution and stirred thoroughly at 65 °C for 2 h. The whole process was repeated three times and the system changed from dark brown to light brown. Hemicellulose was removed from corncob by alkaline treatment. After repeating the operation, the system was cooled to room temperature and a yellow solid was obtained after filtration. Then, the solid was washed with water and ethanol to neutral. Finally, a yellow-brown powder was obtained by drying at 50 °C to constant weight.

The dried powder was added to 250 mL mixed solution of 15.5 g/L NaClO_2_ and 31 mL/L glacial acetic acid and fully stirred at 85 °C for 2 h. The process was repeated three times and the system changed from yellow-green to light yellow. Lignin was removed from corncob by acid treatment. After repeating the operation, the system was cooled to room temperature and a white solid was obtained after filtration. Then, the solid was washed with water and acetone to neutral. Finally, a white powder was obtained by drying at 50 °C to constant weight.

### 2.3. Dissolution of Cellulose

Under the protection of N_2_, 5 g cellulose was added to a reaction flask which contained 100 mL DMAc. The mixture was mechanically stirred at 120 °C for 2 h. The system presented as a white turbid. then, the system began to cool slowly, and 10 g licl was added when it was reduced to 110 °c. The system was stirred while slowly reaching room temperature, until the system was homogeneous. The viscous solution was centrifuged (10,000 rpm; 5 min) to retain the transparent supernatant. DMAc/LiCl can break the hydrogen bond interactions between cellulose molecules to achieve dissolution [23,24]. This causes the cellulose molecular chain to stretch out, enhancing reactivity.

### 2.4. Synthesis of Cellulose Initiator (Cel-Init)

In total, 20 mL DMAc was added to the cellulose solution and stirred to a uniform state to reduce the viscosity of the system. This can increase the fluidity and uniformity of the subsequent reaction. In an ice water bath, 33.4 mL triethylamine (8/3 molar equivalents relative to cellulose hydroxyl groups) was added as acid scavenger and stirred until uniform. While stirring, 18.2 mL 2-chloropropionyl chloride (2 molar equivalents to hydroxyl groups) was slowly added dropwise to the system. This can control the degree of the reaction, to prevent the occurrence of a crosslinking side reaction. By slowly stirring at 0 °C for 11 h, the system gradually became pale yellow in a turbid state.

**Scheme 1:**
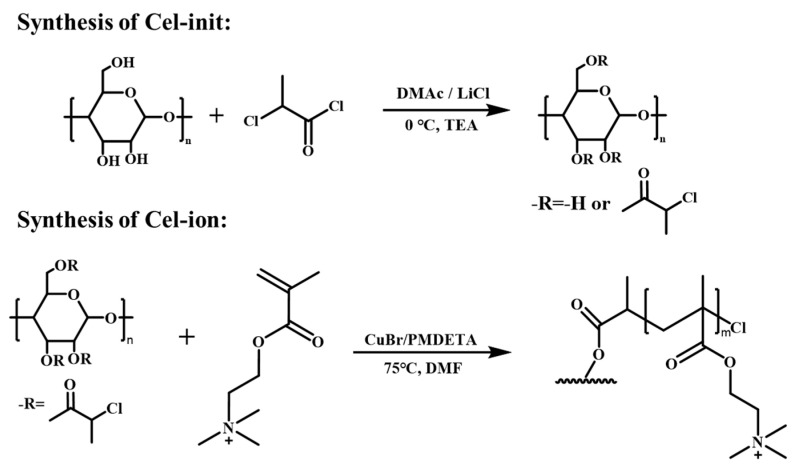
Synthesis scheme of Cel-init and Cel-ion.

After the reaction, the pale yellow turbid liquid was added to water for sedimentation. A yellowish solid was obtained by filtration. The solid was dissolved in THF and dripped into water again for sedimentation. The dissolution–sedimentation–filtration operation was repeated, purifying three times in water and once in ethanol. The final product, which was a cellulose initiator, named Cel-init, was obtained by filtration and vacuum pump drying (Scheme 1).

### 2.5. Synthesis of Ionized Cellulose (Cel-Ion)

Under N_2_ protection, 2 g Cel-init was dissolved in 80 mL DMF in a reaction vial. After the solid was completely dissolved, 2.5 g PMDETA and 32 g DMC monomer were added and stirred until homogeneous and transparent. The solution was bubbled with N_2_ below the liquid level for 15 min, and 2.2 g of CuBr (catalyst) was added under N_2_ protection. The solution was bubbled again for 2 min. Then, a blue solid was obtained after stirring at 75 °C for 5 min. The solid was mixed with water and purified with a dialysis bag (molecular weight cutoff: 8000–14,000 kDa). The product was lyophilized until it became a clear, colorless liquid in the dialysis bag. After the lyophilization of the liquid, a white solid was obtained. This was ionized cellulose, named Cel-ion (Scheme 1).

### 2.6. Fabrication of Cellulose Cation/PDMC Ionogels

Cel-ion (3%, 5%, 7%, and 10% by mass of monomer) was blended with DMC at 50 °C, and then photoinitiator 2959 (1 wt%) was added and stirred until clear. Ionogels were prepared by 365 nm UV light illumination for 5 min, and were named Cel-ion3%-PDMC, Cel-ion5%-PDMC, Cel-ion7%-PDMC, and Cel-ion10%-PDMC.

### 2.7. Characterization Methods

Structural Characterization: The samples were analyzed using a TENSOR II Fourier-transform infrared spectrometer (FTIR) from Bruker Co. (Billerica, MA, USA), in attenuated total reflectance (ATR) mode, with a wavenumber range of 4000–500 cm^−1^ and a resolution of 4 cm^−1^. Nuclear magnetic resonance (^1^*H* NMR) was recorded on an AVANCE III HD 500 MHz spectrometer from Bruker Co. (Billerica, MA, USA). Cel-init was dissolved by deuterated DMSO-d_6_. Cel-ion and ionogel were dissolved by D_2_O. X-ray diffraction (XRD) patterns were obtained using a SmartLab SE diffractometer from Rigaku Co. (Tokyo, Japan), with a scanning angle range of 5–80° and a scan rate of 10°/min.

Thermal Property Analysis: Thermogravimetric analysis (TGA) was carried out using a STA200 thermogravimetric analyzer from HITACHI Co. (Tokyo, Japan), to test the thermal stability of cellulose, Cel-init, and Cel-ion, using N_2_ as a protective gas, with a temperature range of 30–500 °C and a heating rate of 10 °C/min. Due to the high chlorine content in Cel-init, a separate instrument was utilized for gel characterization.

Surface Morphology, Elemental Analysis, Molecular Weight, and Viscosity Characterization: Surface morphology was observed using an Apreo S HiVac scanning electron microscope (SEM) from Thermo Fisher Scientific (Waltham, MA, USA). Samples were cryo-fractured in liquid nitrogen, the water was removed by freeze-drying, and the microstructure of the sample was observed after gold spraying. The elemental content of C, H, and N was determined using a Unicube elemental analyzer from Elementar Analysensysteme GmbH (Frankfurt, Germany). The molecular weight of corncob cellulose was determined via a PL-GPC50 gel permeation chromatography (GPC) from Agilent Technologies Co. Ltd. (Santa Clara, CA, USA), with DMF as the mobile phase. The viscosity changes in the cellulose solution, Cel-ion solutions, and Cel-ion-PDMC gel precursor solution with temperature were characterized by a DV-II+Pro rotational viscometer from AMETEK Brookfield (Middleborough, MA, USA).

Ionogel Property Characterization: The thermal stability of the ionogels was analyzed using a TG 209 F1 thermogravimetric analyzer from NETZSCH-Gerätebau GmbH (Bavaria, Germany), under N_2_ atmosphere with a temperature range of 30–600 °C and a heating rate of 10 °C/min. Differential scanning calorimetry (DSC) was performed using a Q20 instrument from TA Instruments (Newcastle, DE, USA), with a nitrogen flow rate of 100 mL/min, a heating rate was 10 °C/min, and a test temperature range of −20 to 20 °C. A LAMBDA 1050+ UV–vis spectrophotometer from PerkinElmer (Waltham, MA, USA), was used to test the optical transmittance of 1 mm thick gel samples. An Attension theta optical tensiometer from Biolin Scientific AB (Gothenburg, Sweden), was used to characterize the hydrophobicity of the gel samples.

Mechanical properties were characterized using a DR-507AS universal tester of Dongri Instrument Co. (Dongguan, China). The gel was prepared into a dumbbell-shaped spline, size model 4, at a thickness of 2 mm. The test was carried out at 25 °C with a stretching speed of 50 mm/min, and five parallel tests were repeated for each group of samples.

Adhesive properties were tested using toughened glass (6 mm × 10 mm × 60 mm) as the bonding material. The precursor solution of the gel was dropped between the two overlapping glass pieces, and the 365 nm UV light was irradiated for 60 s. The samples were tested using a universal testing machine at 25 °C, with a tensile speed of 50 mm/min. Five parallel tests were repeated for each group of samples.

Water Content and Retention Analysis: Water Content Test: Ionogel films (10 × 10 × 1 mm) were prepared and the initial mass recorded as *M*_0_ (g). After freeze-drying to constant weight, the mass was recorded as *M*_1_ (g). Each group of samples was tested three times, and the final moisture content was averaged. The moisture content (*G*, %) formula of the gel is as follows:

(1) $$G=\frac{M_{0}-M_{1}}{M_{0}}\times 100\%$$

Water Retention Test: Ionogel films (10 mm × 10 mm × 1 mm) were prepared and the initial mass recorded as *m*_0_ (g). The gels were placed in air and a sealed environment, respectively, and the mass on the *n*-th day was recorded as *m_n_* (g). Each group of samples was tested three times, and the final water retention was averaged. The water retention rate (*W*, %) formula of the gel is as follows:

(2) $$W=\frac{m_{0}-m_{n}}{m_{0}}\times 100\%$$

Sensing Performance and Electrocardiogram (ECG) Characterization: Sensing Performance Test: The 2611B digital source table from Tektronix (Solon, OH, USA), was used for testing. The two ends of the gel were connected to the source table with wires, and the gel was fixed on the fixture of the universal testing machine. The tensile state of the gel was controlled by the universal testing machine. The specific stretching speed and strain parameters are detailed in the text. The electrical signal of the gel was recorded by the digital source table. The initial resistance (*R*_0_, Ω) and resistance under strain (*R*, Ω) were measured. The formula of the relative resistance changing rate Δ*R*/*R*_0_ (%) is as follows:

(3) $$\frac{∆R}{R_{0}}=\frac{R-R_{0}}{R_{0}}\times 100\%$$

The strain of the gel is *ε* (%) and the gauge factor (*GF*) formula is as follows:

(4) $$GF=\frac{∆R}{R_{0}}\times \frac{1}{\varepsilon }$$

ECG Test: The prepared gel replaced the commercial gel and was pasted to the wrist and ankle. A PC-80D ECG detector from Creative Industry Co. (Shenzhen, China) was used to detect the ECG curve.

## 3. Results and Discussion

### 3.1. Structural Characterization of Corncob Cellulose and Its Derivatives

**Figure 2:**
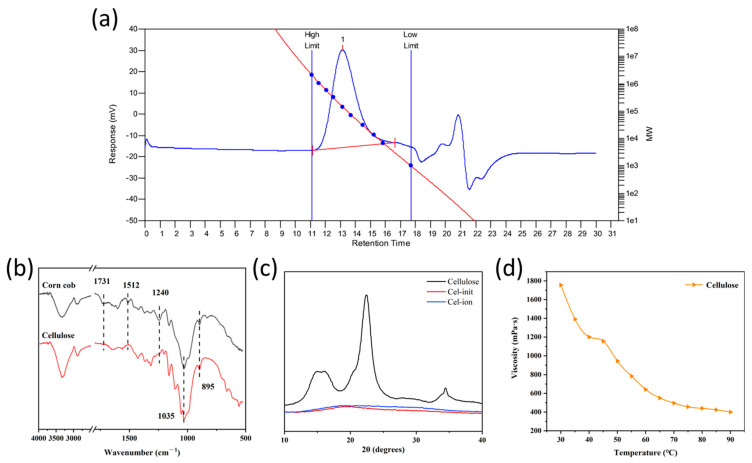
(**a**) GPC curve of corncob cellulose. (**b**) FT-IR spectra of corncob powder and extracted product. (**c**) XRD patterns of cellulose, Cel-init, and Cel-ion. (**d**) Temperature-dependent viscosity curve of cellulose solution in DMAc/LiCl.

GPC analysis revealed that acid-base-treated cellulose retained partial structural integrity. The number-average molecular weight (Mn) was 63,000, the weight-average molecular weight (Mw) was 160,000, and the polydispersity (PDI) was 2.5 (Figure 2a). Meanwhile, the FT-IR analysis of corncob powder and extracted product (Figure 2b) confirmed the effective removal of hemicellulose and lignin. The characteristic peaks of hemicellulose at 1731 cm^−1^ and lignin at 1512 cm^−1^ and 1240 cm^−1^ disappeared, and only the characteristic peaks of cellulose at 1035 cm^−1^ and 895 cm^−1^ were retained. The XRD patterns (Figure 2c) exhibited characteristic cellulose I crystalline with diffraction peaks at 14.6°, 16.2°, 22.6°, and 34.0°. This further confirmed the successful cellulose extraction. The viscosity characterization of the cellulose solution (5 wt% in DMAc/LiCl; Figure 2d) indicated that viscosity was negatively correlated with temperature and demonstrated that the extracted corncob cellulose was well dissolved in the DMAc/LiCl solution and had good fluidity, which provides a basis for chemical modification.

**Figure 3:**
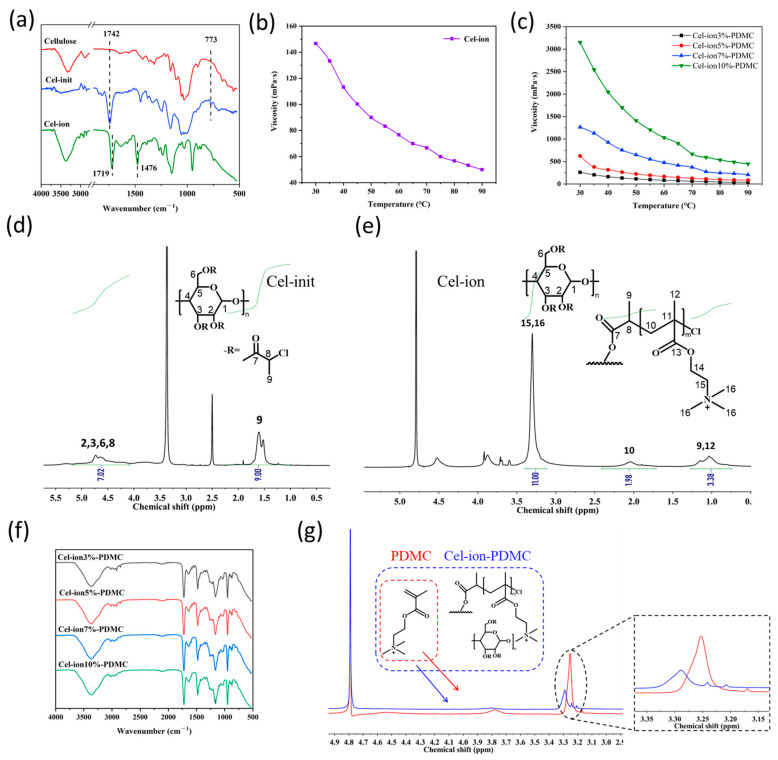
(**a**) FT-IR spectra of cellulose before and after modification. (**b**) Temperature-dependent viscosity curve of Cel-ion solution. (**c**) Temperature-dependent viscosity curves of gel precursor solutions. (**d**) ^1^H NMR spectrum of Cel-init. (**e**) ^1^H NMR spectrum of Cel-ion. (**f**) FT-IR spectrum of ionogel. (**g**) ^1^H NMR spectrum of PDMC and Cel-ion-PDMC.

For Cel-init, the stretching vibration peaks of C-Cl (773 cm^−1^) and C=O (1742 cm^−1^) in the FT-IR spectrum of Cel-init (Figure 3a) strongly prove the successful synthesis of the initiator. Meanwhile, the peak at 2.25–1.00 ppm attributed to H9 (9H, -CH(Cl)C**H**_3_) and the peak at 5.25–4.00 ppm attributed to H2, H3, H6, and H8 (7H, -C**H**(Cl)CH_3_, -C**H**_2_OR, -C**H**(OR)C**H**(OR)-) appeared in the ^1^H NMR spectrum of Cel-init (Figure 3d). This proved that all hydroxyl groups on cellulose were modified into ATRP initiating groups. The XRD analysis (Figure 2c) further indicated the complete loss of crystalline peaks in Cel-init, consistent with its full modification. Elemental analysis (Table 1) revealed that compared to cellulose, the C and H ratios in Cel-init both decreased. This was attributed to the introduction of Cl during the chemical modification. Computational analyses further confirmed that the three hydroxyl groups on the cellulose unit were involved in the reaction.

The FT-IR spectrum of the Cel-ion revealed a characteristic absorption peak at 1719 cm^−1^ corresponding to the C=O stretching vibration. The peaks at 2914 cm^−1^ and 1476 cm^−1^ were attributed to the C-H stretching vibration and bending vibration of the quaternary ammonium groups (Figure 3a). The ^1^H NMR spectrum of Cel-ion (Figure 3e) displayed a peak at 3.40–3.20 ppm, which was assigned to nine protons (16) from three methyl groups in the quaternary ammonium structure, and two protons (15) from adjacent methylene groups. The peaks at 2.20–1.80 ppm corresponded to two methylene protons (10) in the polymer backbone. The signals between 1.25 and 0.75 ppm originated from methyl protons in both the polymer (9) and cellulose initiator structure (12). The quantitative analysis of integrations from ^1^H NMR indicated that eight DMC monomers were grafted onto each cellulose initiator unit. The XRD analysis (Figure 2c) further showed the complete loss of crystalline peaks in Cel-ion, consistent with its successful preparation. Elemental analysis (Table 1) confirmed successful ionic modification with a 4.42% nitrogen content in Cel-ion, and further calculations indicated that the grafting ratio was 1:9. These results collectively suggest an approximate grafting proportion of 1:8–1:9 for cellulose–polymer conjugation. The viscosity curve of the Cel-ion solution (10 wt% in water) (Figure 3b) demonstrated low viscosity and excellent fluidity. Furthermore, the viscosity of the DMC solution (Figure 3c) increased with higher Cel-ion content. Meanwhile, viscosity and temperature showed a significant negative correlation. This indicated Cel-ion compatibility with ionic liquid DMC monomers.

In the FT-IR spectrum of the ionogel (Figure 3f), the peak at 2961 cm^−1^ corresponds to the stretching vibration of the C-H in quaternary ammonium salts. The peak at 1164 cm^−1^ is attributed to the combined stretching vibration of C-N and C-O in PDMC. In the ^1^H NMR spectrum of Cel-ion-PDMC (Figure 3g), the peak at 3.25 ppm is attributed to the three protons of quaternary ammonium salts. Upon complexation with ionized cellulose, this peak exhibited a downfield shift. This indicated the presence of ionic bond interactions between PDMC and ionized cellulose, which perturbed the electronic environment of the hydrogen atoms on the quaternary ammonium group. Ionic interactions drive the structural reorganization during ionogel formation, thereby elucidating the mechanism of ionogel formation.

### 3.2. Thermal Property Characterization

**Figure 4:**
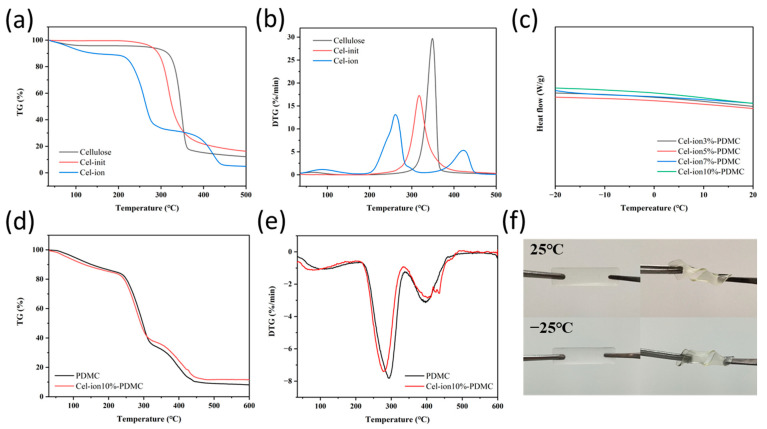
TGA of cellulose, Cel-init, and Cel-ion: (**a**) TG and (**b**) DTG diagrams. (**c**) DSC curves of Cel-ion-PDMC gels. TGA of PDMC and Cel-ion10%-PDMC: (**d**) TG and (**e**) DTG diagrams. (**f**) Appearance and deformation of Cel-ion10%-PDMC at 25 °C and −25 °C (ionogel size is 45 mm × 12 mm × 2 mm).

The TGA (Figure 4a) and DTA (Figure 4b) spectra of cellulose and its derivatives indicate that the weight loss temperatures of cellulose and Cel-init are 348 °C and 317 °C, respectively. This means that the thermal stability of the esterified cellulose initiator decreased. The reason may be the carbon–chlorine bond on the tertiary carbon adjacent to the carbonyl group, which is prone to cleavage, generating small molecules. These molecules further catalyze sample decomposition. The significant reduction in crystallinity after cellulose modification into a macromolecular initiator also contributes to the lower pyrolysis temperature. For Cel-ion, the 10% mass loss observed between 30 and 150 °C is caused by its hygroscopicity. A 60% mass loss was observed at 261 °C, corresponding to the decomposition of the quaternary ammonium salts in PDMC and the cleavage of ester bonds. The mass loss at 422 °C was due to the pyrolysis and carbonization of the cellulose backbone, and further thermal decomposition of earlier degradation products. Notably, the carbon and inorganic salt residues generated during PDMC pyrolysis may coat the cellulose backbone, delaying its thermal degradation process. This shielding effect results in the increased thermal degradation temperature of the ionized cellulose backbone.

The TGA (Figure 4d) and DTA (Figure 4e) analyses of Cel-ion-PDMC gels show that compared to the pure PDMC, the thermal degradation temperature decreased slightly from 292 °C to 278 °C. The DSC curve of Cel-ion-PDMC gels (Figure 4c) demonstrates no endothermic or exothermic peaks between −20 °C and 20 °C. This indicates the absence of phase transitions in this thermal regime. Under 25 °C and −25 °C conditions (maintained for 12 h; Figure 4f), the gel remained transparent and deformable at both 25 °C and −25 °C. These findings collectively demonstrate the gel’s superior temperature adaptability.

### 3.3. Surface Morphology Characterization

**Figure 5:**
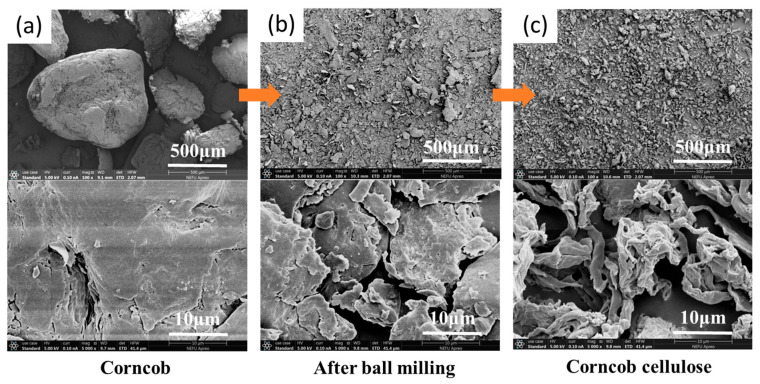
SEM images of (**a**) corncob, (**b**) ball-milled corncob, and (**c**) corncob cellulose.

**Figure 6:**
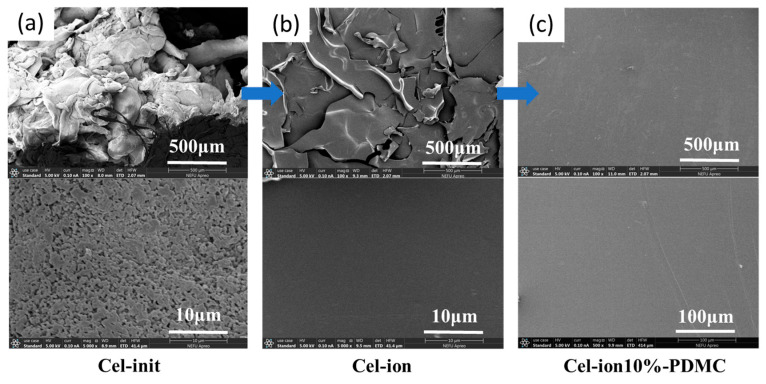
SEM images of (**a**) Cel-init, (**b**) Cel-ion, and (**c**) Cel-ion-PDMC.

The SEM images reveal that the raw corncob particles exhibited diameters around 500 μm (Figure 5a). The low specific surface area results in limited reaction accessibility and makes them unsuitable for direct cellulose extraction. After ball milling treatment, the corncob particles were reduced to 10–50 μm diameters (Figure 5b), and cellulose extraction efficiency was enhanced. The extracted cellulose exhibited granular and rod-like morphologies (Figure 5c). Triethylamine hydrochloride and other water-soluble byproducts remained in the Cel-init crude product. These byproducts were subsequently removed through aqueous washing and formed porous structures on the solid product surface (Figure 6a). After polymerization, the cationic branched cellulose underwent self-aggregation and chain association through various weak intermolecular interactions, finally forming smooth lamellar structures (Figure 6b). Lyophilized Cel-ion10%-PDMC (Figure 6c) displayed a brittle, flat surface due to the reduced intermolecular distances and enhanced interactions.

### 3.4. Transparency, Hydrophilicity, Mechanical Properties, Water Content, and Retention

**Figure 7:**
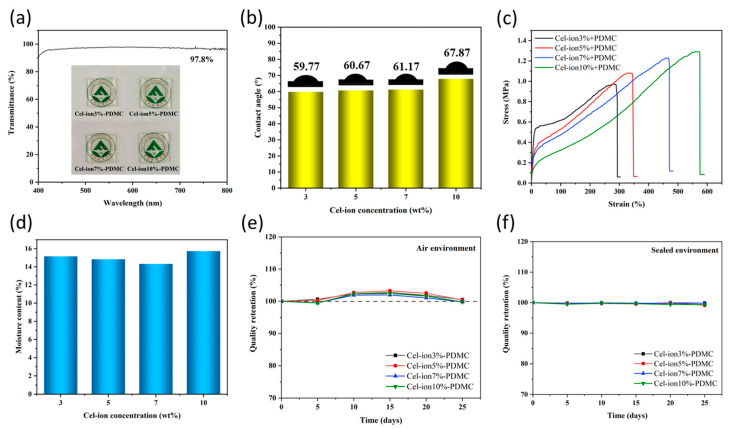
Characterization of Cel-ion-PDMC ionogels. (**a**) Gel photograph and light transmittance curve (ionogel size is 20 mm × 20 mm × 1 mm). (**b**) Contact angle characterization. (**c**) Stress–strain curves. (**d**) Water content analysis. Mass change rate diagram of the gel: (**e**) in the air environment and (**f**) in the sealed environment.

The ionogels exhibited high transparency (97.8%, UV Transmittance), so the university emblem was easily observed through the gels (Figure 7a). The contact angle measurements (Figure 7b) demonstrate a negative correlation between the hydrophilicity of the ionogels and the content of ionized cellulose. This phenomenon is primarily attributed to the intrinsic nature of cellulose ionic brushes. The structural constraints (steric hindrance effects) result in restricted swelling and increased backbone rigidity, compared to linear polymers with the same ionic groups. The steric hindrance hinders the penetration of water molecules into the interior of the polymer and inhibits the swelling capacity of the material. Moreover, the steric hindrance of ionized cellulose results in a denser and more rigid polymer network.

The stress–strain curves of the ionogels (Figure 7c) revealed that the mechanical properties of the gels correlate to the Cel-ion content. The tensile strength and elongation at break were 1.28 MPa and 573%, respectively. The amount of Cel-ion had little effect on the ionogel’s water content, which remained at approximately 15% (Figure 7d). Under ambient conditions (20 °C; 30% humidity), the ionogel exhibited relatively stable mass variation in air, demonstrating good water retention capacity (Figure 7e). The mass remained consistently stable in sealed environments. These indicated that the ionogel possesses satisfactory storage stability in atmospheric and confined conditions over 25 days (Figure 7f).

### 3.5. Adhesive and Cohesive Performance

**Figure 8:**
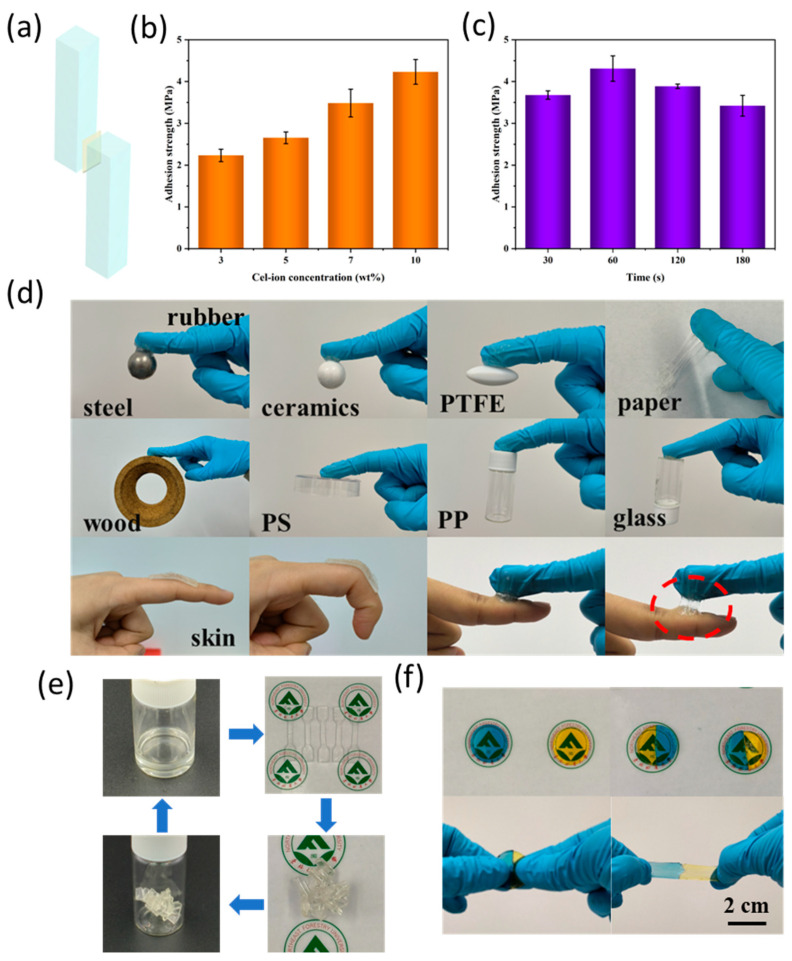
(**a**) Schematic diagram for adhesion test of glass. (**b**) Adhesive strength of gel with different Cel-ion content to glass. (**c**) Adhesive strength of Cel-ion10%-PDMC to glass under different UV irradiation time. (**d**) Adhesion of Cel-ion3%-PDMC gel to rubber, stainless steel, ceramic, PTFE, paper, wood, polystyrene, glass, polypropylene, skin, and skin conformability. (**e**) Schematic of gel recyclability and reprocessability. (**f**) Schematic of the self-healing process.

The ionogel’s bonding strength was tested using a lap shear configuration with two glass strips (Figure 8a). The bonding strength diagram under 60 s UV illumination (Figure 8b) increased from 2.23 MPa to 4.23 MPa with higher Cel-ion content. This demonstrates that ionogels exhibited stronger non-covalent interactions with the glass surface. The increased Cel-ion content enhanced the cohesive strength of the ionogels, thereby improving bonding performance. Optimal curing occurred at 60 s UV exposure (Figure 8c). This indicates that too short a time leads to a low degree of polymerization and weak cohesive strength. Longer times result in too long polymer chains, with less contact between the ions on the polymer and the glass substrate. Adhesion tests of Cel-ion3%-PDMC on diverse substrates (stainless steel, ceramic, PTFE, paper, wood, polystyrene, glass, polypropylene, and skin) demonstrated its excellent adhesion properties (Figure 8d). The material showed good properties, indicating promising potential for human joint monitoring, particularly in finger joint flexion detection applications.

### 3.6. Recyclability and Self-Healing Properties

Since ionogels were achieved by physical crosslinking via ionic interactions and hydrogen bonding. There were no permanent chemical crosslinks in the ionogels. Therefore, ionogel samples can be crushed and dissolved in water, and then be re-dehydrated to form new shapes (Figure 8e), showing excellent recyclability and reprocessability. The cross-section of methyl orange- and methylene blue-stained ionogels, showed self-healing capability after 20 min of room-temperature contact, with tensile images (Figure 8f) demonstrating remarkable self-healing properties. These self-healing properties significantly enhance the service life and damage resistance of ionogels as flexible sensors, providing better reliability for long-term applications.

### 3.7. Sensing Performance Characterization

**Figure 9:**
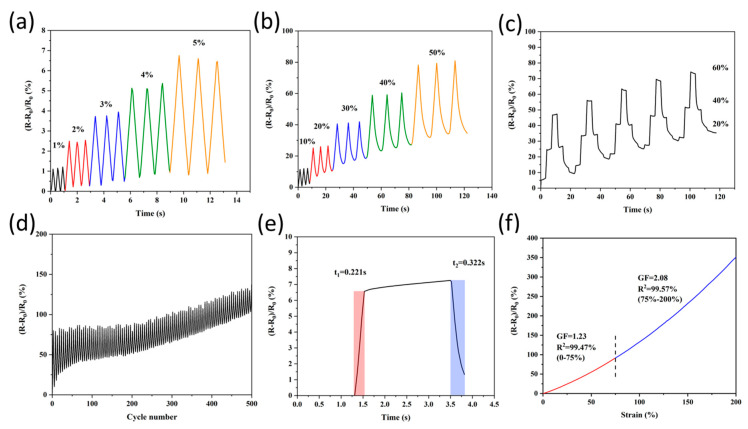
Sensing performance of the ionogel. (**a**) Small strain response (1–5%). (**b**) Large strain response (10–50%). (**c**) Resistance changes under 20%, 40%, and 60% strain. (**d**) Results of 500-cycle tests. (**e**) Response and recovery times. (**f**) Sensitivity test.

The ionogel (Cel-ion3%-PDMC) was systematically evaluated as a flexible sensing detector with copper electrodes. The ionogel exhibited excellent linearity and reproducibility in the electrical signal-strain response within small strain ranges (1–5%) (Figure 9a), highlighting its superior electrical sensitivity. Ion transport pathways elongate with strain increase, leading to higher resistance. Conversely, shortened pathways reduced resistance during the recovery phase, enabling the electrical signal to respond to strain. This result indicates that the ionogel has excellent small strain detection capability. For larger strains (10–50%) (Figure 9b), the relative resistance change retained reproducibility. However, it deviated from baseline during the recovery phase, attributed to hysteresis-induced sensitivity reduction. Electrical signal detection under 20%, 40%, and 60% strains revealed relative linear and repeatability (Figure 9c). The gel’s adaptability and stability for 20–60% strain monitoring were verified. Cyclic strain testing (500 cycles; 0–50% strain) showed stable resistance variation amplitudes (Figure 9d), suggesting that the ionogel has potential for repeated applications. The gel demonstrated rapid response and recovery times of 0.211 s and 0.322 s, under a stretching rate of 500 mm/min (Figure 9e), ensuring real-time strain detection.

**Figure 10:**
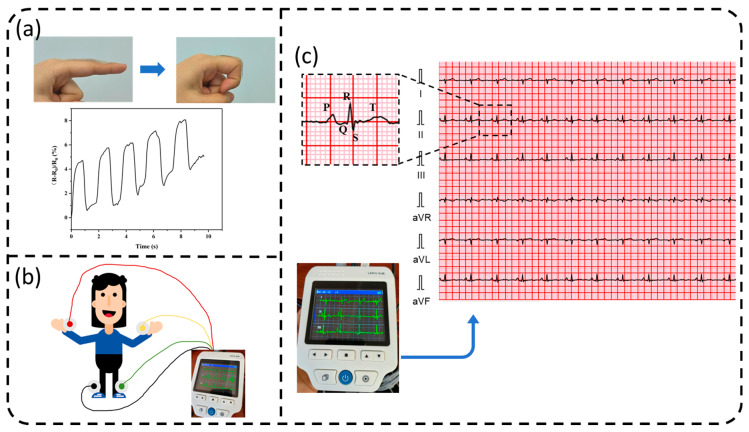
(**a**) Sensing test diagram of finger joint bending. (**b**) Connection diagram of electrocardiogram test. (**c**) ECG test curve.

The sensitivity analysis (Figure 9f) demonstrated gauge factors (GFs) of 1.23 (*R*^2^ = 99.47%) for 0–75% strain and 2.08 (*R*^2^ = 99.57%) for 75–200% strain. Practical applications were demonstrated through human motion monitoring (e.g., finger bending) (Figure 10a) and ECG signal acquisition (Figure 10b,c). The ECG curves exhibited distinct PQRST waveforms, confirming the ionogel’s reliability in health and motion sensing.

## 4. Conclusions

This study presents an innovative strategy to convert corncob cellulose into high-performance ionogels through a combination of macromolecular initiator modification on cellulose and UV-initiated polymerization. Comprehensive structural and thermal analyses, including FTIR, ^1^H NMR, XRD, TGA, and SEM, confirmed the successful formation of a 3D network gel with enhanced mechanical properties (1.28 MPa tensile strength with an elongation at break of 573%) and robust thermal stability (initial decomposition temperature >250 °C). Furthermore, the ionogel sensor exhibited universal adhesion, effective self-healing, and remarkable strain-sensing performance (a *GF* of 1.23–2.08), enabling the accurate monitoring of human joint motion and reliable ECG signal acquisition. By integrating agricultural waste valorization with advanced material design, this work aligns with green chemistry principles and offers a sustainable alternative to petroleum-based sensors, thereby broadening the application scope of biomass-derived smart materials in flexible electronics and beyond.

## Figures and Tables

**Table 1:** Elemental composition.

Samples	C (%)	H (%)	N (%)
Cellulose	42.22	5.994	0.00
Cel-init	41.75	4.365	0.00
Cel-ion	36.05	6.413	4.42

## Data Availability

Data will be made available on request.
